# Developmental Stage-Driven Niche Differentiation and Assembly of Rhizosphere and Endophytic Bacterial Communities in *Helianthus annuus* Under Saline–Alkaline Stress

**DOI:** 10.3390/microorganisms14020404

**Published:** 2026-02-08

**Authors:** Bo Liu, Tingting Lu, Ting Yao, Xiujuan Zhao, Lihua Yang

**Affiliations:** 1 Laboratory for Environmental Microbiology and Biotechnology in Arid and Cold Regions, College of Life Sciences, Inner Mongolia Agricultural University, Hohhot 010011, China; 2 Key Laboratory of Biomanufacturing in Inner Mongolia Autonomous, Hohhot 010011, China

**Keywords:** plant–microbe interactions, community assembly, ecological filtering, microbial co-occurrence networks, nitrogen cycling potential

## Abstract

Soil salinization severely constrains agricultural productivity, while root-associated microbiota contribute to plant adaptation to saline–alkali stress. However, developmental assembly dynamics of rhizosphere and root endosphere bacterial communities remain insufficiently characterized in irrigation-driven saline–alkali agroecosystems such as the Hetao Plain of northern China. Here, *Helianthus annuus* plants were sampled at seedling, squaring, and flowering stages, and rhizosphere and root microbiota were analyzed using high-throughput amplicon sequencing integrated with soil physicochemical measurements, beta nearest taxon index–based community assembly inference, and co-occurrence network analysis. The rhizosphere maintained higher diversity, broader taxonomic heterogeneity, and persistently complex interaction networks, whereas the root endosphere exhibited progressive diversity reduction and compositional convergence during plant development. Developmental progression drove contrasting successional trajectories, with increasing rhizosphere complexity and endophytic convergence toward a Proteobacteria-dominated core, particularly *Pseudomonas*. Beta nearest taxon index analysis indicated mixed stochastic and dispersal-related processes in the rhizosphere but drift-dominated assembly in late-stage roots. Functional predictions revealed enhanced nitrogen-related metabolic potential during flowering, coinciding with enrichment and network centrality of *Pseudomonas*. These findings demonstrate stage-dependent spatial reorganization of sunflower root microbiomes under saline–alkali stress and provide a framework for identifying functionally relevant microbial groups for targeted microbiome-based agricultural management.

## 1. Introduction

Soil salinization has become a major barrier to global agricultural sustainability, affecting an estimated 7–8% of the world’s land area and continuing to expand rapidly. High salt and alkali levels can cause osmotic stress, ion toxicity, root damage, and reduced nutrient availability, which collectively inhibit crop growth and weaken soil microbial processes [1,2]. In the arid and semi-arid regions of northern China, saline–alkali soils are widespread and significantly limit the productivity of grain and oil crops, including in typical areas such as the Hetao irrigation district of Inner Mongolia [3]. Addressing soil degradation in these regions is therefore essential for improving agricultural output.

*Helianthus annuus* L. is a key cash and oilseed crop in northern saline–alkali areas of China. It exhibits relatively strong tolerance to salt and alkali stress and plays an important role in local agricultural systems. Its well-developed root system, together with effective ion regulation and nutrient redistribution, makes *H. annuus* one of the most suitable crops for saline–alkali soils [4]. Studies have shown that rhizosphere and endophytic microorganisms can alleviate saline–alkali stress through multiple mechanisms, including improving nutrient availability, regulating ion uptake, promoting the synthesis of stress-related metabolites, and enhancing antioxidant systems [5,6,7]. Although *H. annuus* exhibits inherent salt-tolerance traits, how its rhizosphere and endophytic microbial communities are differentially assembled and functionally contribute to adaptation across developmental stages in saline–alkaline soils remains unresolved, limiting mechanistic insight into plant–microbe interactions and their application in saline agriculture [8,9,10].

Generally, the rhizosphere hosts diverse microbial communities with functional redundancy [11], while the root endosphere is shaped by strong host filtering, allowing only a small set of beneficial or protective taxa to colonize [12]. In addition to these well-defined rhizosphere and endophytic compartments, commensalist and facultative microorganisms that can exist either as epiphytes on the root surface or as endophytes within root tissues may also contribute to plant performance under saline–alkali conditions [13]. Understanding how these two microbial niches are structured in saline–alkali environments is therefore crucial for clarifying the microbial basis of *H. annuus* salt tolerance. On the other hand, plant developmental stage is another major driver of root microbiota dynamics. Changes in root exudation, nutrient requirement, tissue structure, and physiological state during growth can strongly influence microbial recruitment, colonization, and interactions [14]. However, although rhizosphere and root-associated bacterial microbiota of *H. annuus* and other crops grown in saline soils have been widely investigated [5,9], their developmental dynamics and niche differentiation under irrigation-induced saline–alkaline conditions remain insufficiently quantified. In the Hetao Irrigation District of Bayannur (Inner Mongolia), long-term irrigation with Yellow River water has resulted in extensive secondary salinization, generating a persistent and region-specific salt stress environment [15]. Under this context, the extent to which *H. annuus* rhizosphere and root bacterial communities exhibit stage-dependent divergence, follow distinct assembly tendencies, and show differential enrichment of functional groups has not been fully resolved. These knowledge gaps limit our understanding of the microbial ecology of crops in saline–alkali soils and hinder the development of targeted synthetic microbial communities (SynComs) for improving salt tolerance and crop productivity in these challenging environments [16,17].

To address these gaps, we conducted a field experiment in the Hetao Plain, Inner Mongolia, to investigate the rhizosphere and root microbiomes of *H. annuus* grown under saline–alkali conditions. Using high-throughput amplicon sequencing, soil physicochemical and enzyme activity analyses, βNTI-based community assembly modeling, functional prediction, and co-occurrence network analysis, we modeled the microbial structure, function, and plausible assembly mechanisms across two spatial niches (rhizosphere vs. root endophytic) and three developmental stages (seedling stage, squaring stage, flowering stage). The objectives were to: (1) describe the spatial differentiation of rhizosphere and root endophytic bacterial amplicon communities; (2) reveal stage-driven shifts in bacterial amplicon diversity, composition, and assembly processes; (3) identify key core taxa and predict changing network roles; and (4) assess functional potentials related to salinity adaptation. This study provides new insights into the ecology of root-associated microbiomes in salt-tolerant crops under the region-specific secondary salinization of the Hetao Irrigation District and offers a scientific basis for designing functionally defined *H. annuus* SynComs tailored to the unique irrigation-induced saline–alkaline conditions of this area.

## 2. Materials and Methods

### 2.1. Test Site and Sampling Design

A one-year field experiment was conducted in Bayannur City (Hetao Plain, Inner Mongolia; 40°50′4.628″ N, 107°27′12.035″ E; 1010–1050 masl), an arid-semi-arid area affected by cold continental monsoon. The climate is cold-dry with a mean annual temperature of 6.5–7.5 °C, an annual precipitation of 130–220 mm (70% in July–September) and evaporation of 2000–2400 mm. The soil is Calcic Orthic Solonchaks, with EC_e_ 300–500 μS/cm and pH 8.0–8.5 [18]. The site is cropland under a one-year rotation, with maize being the crop prior to *H. annuus* (cultivar HT661). The plants were grown under local conventional management practices, including basal fertilization with 150 kg ha^−1^ compound fertilizer (N:P:K = 15:15:15), topdressing with 75 kg ha^−1^ urea at the squaring stage, no soil amendments, and flood irrigation with Yellow River water applied at the seedling and squaring stages. Sampling was performed at three key developmental stages of *H. annuus*: seedling (HA1, 25 days after planting, DAP), squaring (HA2, 45 DAP), and flowering (HA3, 65 DAP). For each stage, five plants were randomly selected, and both rhizosphere soil and root tissues were collected. Rhizosphere soil was taken from the soil tightly adhering to the roots. Root samples were washed with sterile PBS to remove residual soil before DNA extraction.

### 2.2. Soil Physicochemical Properties

Rhizosphere soil samples collected at different developmental stages were air-dried at room temperature, ground, and passed through a 2 mm sieve. The measured parameters included soil pH, which were determined in a 1:5 (*w*/*v*) soil–water suspension using a HI-2221 pH meter (Hanna Instruments, Padua, Italy). Soil organic matter (SOM) was determined using the potassium dichromate oxidation–titration method. Soil available phosphorus (P, AP) and available nitrogen (AN) were measured using sodium bicarbonate (NaHCO_3_) extraction and the ferrous sulfate method, respectively [19].

The activities of soil enzymes—including soil catalase (S-CAT), soil alkaline phosphatase (S-AKP/ALP), soil alkaline protease (S-ALPT), soil urease (S-UE), soil dehydrogenase (S-DHA), and soil sucrase (S-SC)—were determined using commercial assay kits (S-CAT: G0303W96; S-AKP/ALP: G0305W48; S-ALPT: G0314W; S-UE: G0301W96; S-DHA: G0307W48; S-SC: G0302W96, Grace Biotechnology Co., Ltd., Suzhou, China) according to the manufacturer’s instructions [20].

### 2.3. DNA Extraction and High-Throughput Sequencing

Environmental DNA from rhizospheric soil samples and clean root samples was extracted using the DNeasy^®^ PowerSoil^®^ Pro Kit (QIAGEN, Germantown, MD, USA) and the FastDNA^®^ Spin Kit for Soil (MP Biomedicals, Irvine, CA, USA), respectively, according to the manufacturers’ protocols. For bacterial community analysis, the V5–V7 hypervariable regions of the 16S rRNA gene in root endophytic samples were amplified using the primer pair 799F (5′-AACMGGATTAGATACCCKG-3′) and 1193R (5′-ACGTCATCCCCACCTTCC-3′), while the V3–V4 regions in rhizosphere samples were amplified using the 338F primer (5′-ACTCCTACGGGAGGCAGCAG-3′) and the 806R primer (5′-GGACTACHVGGGTWTCTAAT-3′) with an ABI GeneAmp^®^ 9700 PCR thermocycler (ABI, Los Angeles, CA, USA). DNA concentration and purity were assessed using a NanoDrop^®^ ND-2000 spectrophotometer (Thermo Scientific Inc., Waltham, MA, USA). Libraries were constructed using a NEXTFLEX^®^ Rapid DNA-Seq Kit (Bioo Scientific, Austin, TX, USA), and high-throughput sequencing was performed on the Illumina MiSeq PE300 platform (Illumina, San Diego, CA, USA) by Shanghai Majorbio Bio-pharm Technology Co., Ltd. (Shanghai, China). Raw sequencing reads were quality-filtered using Fastp (0.23.2) [21] and merged using FLASH (1.2.11) [22]. The optimized sequences were further denoised using the DADA2 (2024) [23] plugin in QIIME2 (2024) [24] to obtain amplicon sequence variants (ASVs). Taxonomic annotation of ASVs was performed using the naive Bayes classifier in QIIME2 against the SILVA database (v138) [25].

### 2.4. Co-Occurrence Network Construction

To examine microbial interactions in the rhizosphere and root niches, co-occurrence networks were constructed at the ASV level. Only ASVs with relative abundance > 0.01% and prevalence > 20% were retained to reduce noise from rare taxa. Spearman correlation coefficients were calculated to assess associations among ASVs, and correlations with |r| > 0.7 and *p* < 0.001 were included in network construction [26]. Network parameters—including the number of nodes and edges, average degree, network density, and modularity—were calculated using the R package ‘igraph’ version 1.5.1 [27,28]. Key taxa and structural features across developmental stages and niches were identified, and networks were visualized using Gephi (version 10.1) [29].

### 2.5. Bioinformatic and Statistical Analyses

Microbial diversity and community structure analyses were also performed using QIIME2 and the Mothur (1.30.2) [30]. Alpha diversity was evaluated using the Shannon and Chao1 indices, and pairwise comparisons between groups were performed using Tukey’s honestly significant difference (HSD) test [31]. Beta diversity was evaluated based on Bray–Curtis dissimilarities and visualized by principal coordinates analysis (PCoA), with differences among developmental stages tested using permutational multivariate analysis of variance (PERMANOVA) [32]. Differentially abundant taxa were identified using LEfSe (LDA > 2.5) and the Kruskal–Wallis test, followed by false discovery rate (FDR) correction for multiple comparisons [33]. Redundancy analysis (RDA) and Spearman’s rank correlation were performed to examine the influence of environmental factors on bacterial community variation, assessing associations between soil physicochemical properties–community structure and bacterial genera abundance–environmental variables [34]. Community assembly processes were inferred by jointly applying the β-nearest taxon index (βNTI) and RC_bray metrics to quantify the relative contributions of deterministic and stochastic processes to the formation of rhizosphere and endophytic communities [35].

## 3. Results

### 3.1. Changes in α-Diversity of Rhizosphere and Root Bacterial Communities

**Figure 1:**
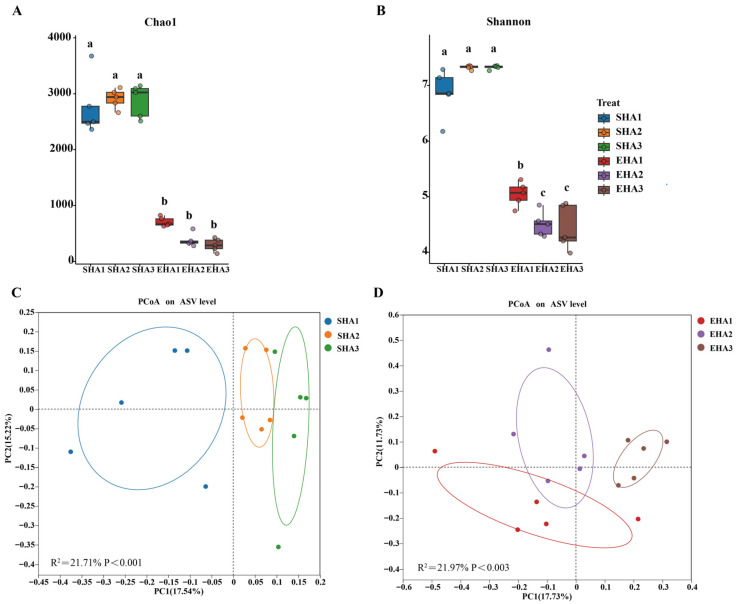
Alpha and beta diversity patterns across *H. annuus* developmental stages in saline–alkaline soil. (**A**,**B**) Alpha diversity (Chao1 richness and Shannon index) of rhizosphere and endophytic communities across developmental stages. Different letters indicate significant differences (LSD test, *p* < 0.05). PCoA based on Bray–Curtis dissimilarity showing stage-dependent shifts in rhizosphere (**C**) and endophytic (**D**) bacterial community structure. Ellipses represent 95% confidence intervals; PERMANOVA indicates significant stage effects.

When sequencing depth reached 20,000 reads, the Chao1 rarefaction curves for all treatments approached a plateau (Figure S1). Alpha-diversity analysis showed that the rhizosphere bacterial community had higher richness and diversity than the root bacterial community at all three developmental stages (Figure 1A,B). In the rhizosphere, the Chao1 index was highest at the squaring stage (SHA2), while the Shannon index reached its maximum at the flowering stage (SHA3). Both indices increased across developmental stages and showed no further increase at the flowering stage.

In the root bacterial community, richness and diversity were consistently lower than those observed in the rhizosphere. Both the Chao1 and Shannon indices decreased across developmental stages (Figure 1A,B). Specifically, the Chao1 index was significantly higher at the seeding stage (EHa1, 707.23) than at both the squaring stage (EHA2, 378.84) and flowering stage (EHa3, 291.4; *p* < 0.05) (Table S1), whereas no significant difference was observed between the EHa2 and EHa3 (Figure 1C).

### 3.2. β-Diversity of Rhizosphere and Root Bacterial Communities

In the rhizosphere, PC1 and PC2 explained 17.54% and 15.22% of the total variation, respectively, and samples from the three growth stages were separated in ordination space (PERMANOVA: R^2^ = 21.97%, *p* < 0.001). In the root endosphere, PC1 and PC2 explained 17.73% and 11.73% of the variation, respectively, with samples from different developmental stages also forming distinct clusters (PERMANOVA: R^2^ = 21.76%, *p* < 0.001, Figure 1C).

The relative contributions of community assembly processes differed between rhizosphere and root endosphere across developmental stages (Figure S2). In the rhizosphere, diffusion limitation and drift were the dominant processes at the seedling stage (SHA1). At the squaring stage (SHA2), the relative contribution of drift increased, while diffusion limitation decreased. At the flowering stage (SHA3), the contribution of drift declined, diffusion limitation increased, and homogeneous diffusion was detected. In the root endosphere, diffusion limitation accounted for 56% of the assembly processes at the seedling stage (EHA1). At the squaring stage (EHA2), drift accounted for 68%, and at the flowering stage (EHA3), drift accounted for 100% of the inferred assembly processes.

### 3.3. Composition and Differential Analysis of Rhizosphere and Endosphere Bacterial Communities

**Figure 2:**
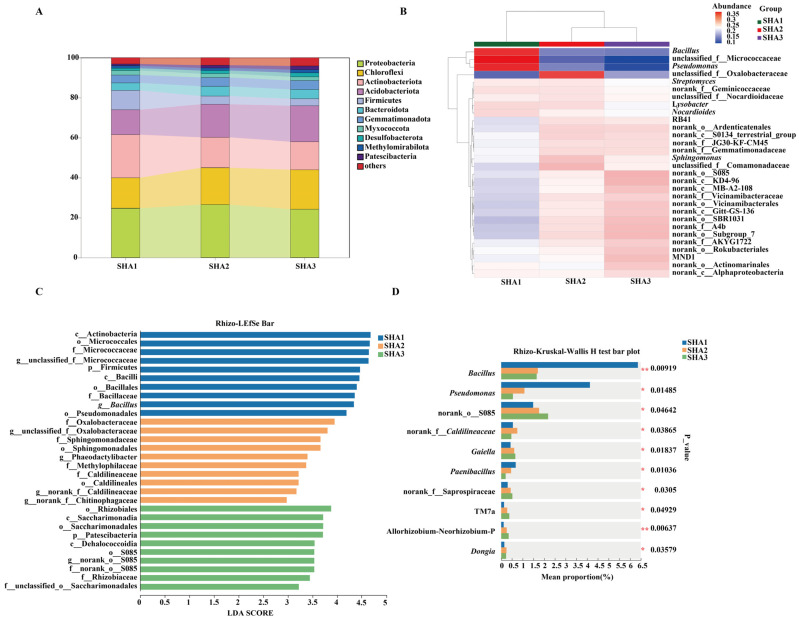
Taxonomic composition and stage-associated bacterial taxa in the rhizosphere across *H. annuus* developmental stages. (**A**) Relative abundance of dominant bacterial phyla in rhizosphere samples at different stages. (**B**) Heatmap showing the relative abundance of the top 30 dominant bacterial genera at the genus level across different stages. (**C**) LEfSe analysis identifying bacterial taxa significantly enriched at each developmental stage (LDA score > 3.5). (**D**) Kruskal–Wallis H test showing genera with significant differences in relative abundance among stages (*p* < 0.05). * indicates *p* < 0.05, ** indicates *p* < 0.01, Bars represent mean relative abundance.

In the rhizosphere soil, 11 bacterial phyla were detected across all developmental stages. The dominant phyla (relative abundance >10%) were Proteobacteria, Chloroflexi, Actinobacteriota, and Acidobacteriota, and their relative abundances varied among stages (Figure 2A). Firmicutes showed higher relative abundance at the seedling stage and lower abundance at the squaring and flowering stages. Genus-level phylogenetic heatmap and hierarchical clustering showed stage-dependent differences in community composition (Figure 2B). *Bacillus* and *Pseudomonas* showed higher relative abundance at the seedling stage, whereas several genera with unresolved taxonomic assignments increased at the squaring and flowering stages. Kruskal–Wallis tests identified *Bacillus*, *Pseudomonas*, *Gaiella*, and *Paenibacillus* as significantly different among developmental stages (*p* < 0.05), with *Bacillus* and *Pseudomonas* showing higher relative abundance at the seedling stage (Figure 2D). LEfSe analysis identified taxa enriched at different stages, including Firmicutes-, Bacilli-, and Pseudomonas-related lineages at the seedling stage (SHA1), Sphingomonadaceae-related taxa at the squaring stage (SHA2), and Patescibacteriota at the flowering stage (SHA3) (LDA > 3.5, *p* < 0.05) (Figure 2C).

**Figure 3:**
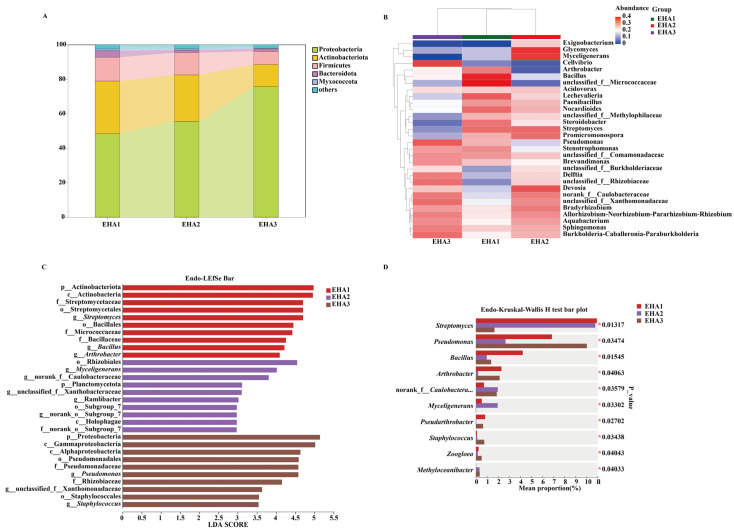
Taxonomic composition and stage-associated bacterial taxa of root endophytic communities at the seedling (EHA1), squaring (EHA2), and flowering (EHA3) stages. (**A**) Relative abundance of dominant bacterial phyla in root endophytic samples. (**B**) Heatmap showing the relative abundance of the top 30 dominant bacterial genera at the genus level across different stages. (**C**) LEfSe analysis identifying bacterial taxa significantly enriched in endophytic communities at different stages (LDA score > 2.5). (**D**) Kruskal–Wallis H test of genera showing significant differences in relative abundance among developmental stages (*p* < 0.05), * indicates *p* < 0.05. Bars indicate mean relative abundance.

In the endosphere, five bacterial phyla were detected, with Proteobacteria and Actinobacteriota accounting for relative abundances above 10% across all stages. The relative abundance of Proteobacteria increased across developmental stages, while that of Actinobacteriota decreased (Figure 3A). At the genus level, *Bacillus* and *Lechevalieria* showed higher relative abundance at the seedling stage (EHA1); *Glycomyces* and *Myceligenerans* increased at the squaring stage (EHA2); and *Pseudomonas* and *Cellvibrio* showed higher relative abundance at the flowering stage (EHA3) (Figure 3B). Kruskal–Wallis tests showed significant differences among stages for *Bacillus*, *Pseudomonas*, and *Streptomyces* (*p* < 0.05) (Figure 3D). *Bacillus* showed the highest relative abundance at the seedling stage, *Pseudomonas* decreased at the squaring stage and increased at the flowering stage, and *Streptomyces* reached its lowest relative abundance at the flowering stage. LEfSe analysis identified stage-enriched taxa in the endosphere, including Actinobacteriota- and Bacillus-related lineages at the seedling stage (EHA1), Rhizobiales- and Caulobacterales-related taxa at the squaring stage (EHA2), and Proteobacteria- and *Pseudomonas*-related taxa at the flowering stage (EHA3) (LDA > 3.5, *p* < 0.05) (Figure 3C).

Venn analysis showed that 693 genera were shared among the three developmental stages in the rhizosphere, with 81, 66, and 98 genera unique to the seedling (SHA1), squaring (SHA2), and flowering (SHA3) stages, respectively. In the endosphere, 218 genera were shared across stages, with 187, 44, and 77 genera unique to EHA1, EHA2, and EHA3, respectively (Figure S3).

### 3.4. Co-Occurrence Network Analysis

**Figure 4:**
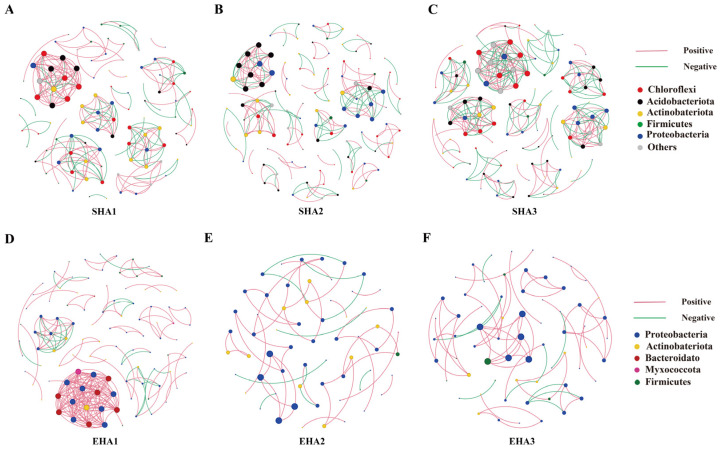
Co-occurrence network structure of rhizosphere and endophytic bacterial communities and variations in soil physicochemical properties across *H. annuus* developmental stages. (**A**–**C**) Co-occurrence networks of rhizosphere bacterial communities at the seedling (SHA1), squaring (SHA2), and flowering (SHA3) stages. (**D**–**F**) Co-occurrence networks of endophytic bacterial communities at the corresponding developmental stages (EHA1–EHA3). In the networks, nodes represent bacterial genera, and node size is proportional to relative abundance. Node colors indicate bacterial phyla. Red and green lines denoting positive and negative correlations, respectively.

In the rhizosphere, network size ranged from 104 to 123 nodes and 205 to 297 edges across stages. The seedling-stage network (SHa1) comprised 104 nodes and 229 edges, with an average degree of 4.404 and a network density of 0.0428 (Figure 4A). At the squaring stage (SHa2), node number increased to 123, while edge number (205), average degree (3.333), and network density (0.0273) decreased (Figure 4B). At the flowering stage (SHa3), network connectivity increased markedly, with 297 edges, the highest average degree (5.211), and the highest network density (0.0461) (Figure 4C). The proportion of negative correlations increased progressively from 35.4% at the seedling stage to 40.0% at the squaring stage and 51.2% at the flowering stage (Table 1).

In contrast, root endophytic networks showed a pronounced contraction during development. The seedling-stage endophytic network (EHa1) contained 103 nodes and 222 edges, with an average degree of 4.311 and a network density of 0.0423 (Figure 4D). At the squaring stage (EHa2), network size declined sharply to 61 nodes and 51 edges, accompanied by a reduction in average degree (1.672) and network density (0.0279) (Figure 4E). The flowering-stage network (EHa3) remained small, with 66 nodes and 70 edges, and showed only a slight increase in average degree (2.121) (Figure 4F). Across all stages, endophytic networks were dominated by positive correlations (80.4–86.9%), while negative correlations accounted for a minor proportion of edges (13.1–19.6%) (Table 1).

Modularity differed markedly between the two niches. Rhizosphere networks showed relatively stable modularity values across stages (0.862–0.908), whereas endophytic networks exhibited substantially higher modularity at the squaring and flowering stages (0.943 and 0.911, respectively), despite their reduced size and connectivity.

### 3.5. Changes in Soil Physicochemical Properties and Enzyme Activities

**Figure 5:**
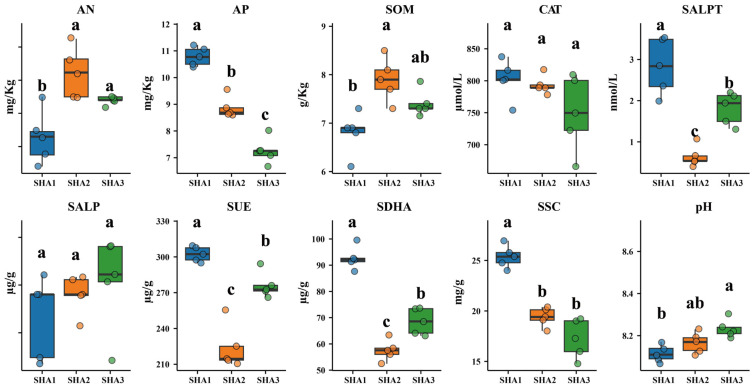
Boxplots showing changes in rhizosphere soil physicochemical properties and enzyme activities across sunflower developmental stages. Parameters include available nitrogen (AN), available phosphorus (AP), soil organic matter (SOM), soil catalase activity (CAT), soil alkaline protease activity (SALPT), soil alkaline phosphatase activity (SALP), soil urease activity (SUE), soil dehydrogenase activity (SDHA), soil sucrase activity (SSC), and soil pH. Different lowercase letters indicate significant differences among developmental stages (LSD test, *p* < 0.05).

As shown in Figure 5, the physicochemical properties and enzyme activities of rhizosphere soil varied across plant growth stages. Soil available nitrogen (AN) and soil organic matter (SOM) reached their highest values at the squaring stage, with AN at 4.22 mg·kg^−1^ and SOM at 7.90 g·kg^−1^, both higher than those at the seedling stage. Available phosphorus (AP) decreased continuously across development, from 10.79 mg·kg^−1^ at the seedling stage to 7.32 mg·kg^−1^ at the flowering stage.

Soil enzyme activities also differed among stages (Figure 5). Soil alkaline protease (SALPT) showed the highest activity at the seedling stage and lower activities at the squaring and flowering stages. Soil urease (SUE) decreased from 302.31 μg·g^−1^ at the seedling stage to 223.81 μg·g^−1^ at the squaring stage and increased to 276.12 μg·g^−1^ at the flowering stage. Soil dehydrogenase (SDHA) exhibited the highest activity at the seedling stage, the lowest at the squaring stage, and higher activity at the flowering stage. Soil sucrase (SSC) decreased from 25.37 mg·g^−1^ at the seedling stage to 17.25 mg·g^−1^ at the flowering stage. Soil pH increased from 8.116 at the seedling stage to 8.166 at the squaring stage and 8.232 at the flowering stage, remaining within the alkaline range. Other soil properties showed no significant differences among stages.

### 3.6. Relationships Between Rhizosphere and Endosphere Bacterial Communities and Environmental Factors

**Figure 6:**
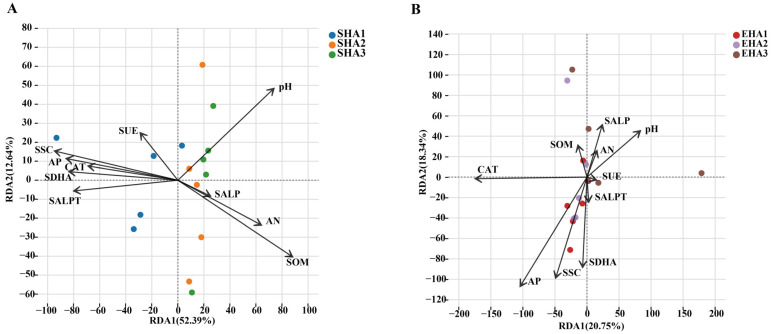
Relationships between soil physicochemical properties and bacterial communities in the rhizosphere (left panels) and endosphere (right panels) across different developmental stages. (**A**,**B**) Redundancy analysis (RDA) showing associations between bacterial community structure and soil properties in the rhizosphere (**A**) and endosphere (**B**). Arrows represent soil physicochemical parameters and enzyme activities, with length and direction indicating correlation strength and direction. Points denote samples from different developmental stages.

Redundancy analysis (RDA) showed contrasting patterns of association between environmental variables and bacterial community composition in the rhizosphere and endosphere (Figure 6). For the rhizosphere community, soil organic matter (SOM; r^2^ = 0.602, *p* = 0.007), available phosphorus (AP; r^2^ = 0.475, *p* = 0.019), soil sucrase activity (SSC; r^2^ = 0.587, *p* = 0.005), soil dehydrogenase activity (SDHA; r^2^ = 0.443, *p* = 0.024), soil alkaline protease activity (SALPT; r^2^ = 0.402, *p* = 0.029), and pH (r^2^ = 0.477, *p* = 0.025) were significantly correlated with community variation (Table S2). For the endophytic bacterial community, the explanatory power of the first two RDA axes for the endophytic bacterial community was relatively lower (Figure 6B). Among the tested variables, only soil catalase activity (CAT; r^2^ = 0.630, *p* = 0.010) and available phosphorus (AP; r^2^ = 0.465, *p* = 0.017) showed significant correlations with community composition (Table S2).

In the rhizosphere, several dominant genera showed significant correlations (*p* < 0.05) with multiple soil properties and enzyme activities. *Pseudomonas* exhibited significant correlations with AP, SALPT, SSC, and pH, while *Bacillus* was significantly correlated with SOM, SDHA, and SSC. Unclassified Micrococcaceae also showed significant correlations with most measured variables, except CAT and SALPT (Figure S4A). In the endosphere, correlation coefficients between dominant genera and environmental variables were generally lower (r < 0.5). However, several abundant taxa, including unclassified Caulobacteraceae, Streptomyces, Arthrobacter, and *Bacillus*, exhibited significant correlations (*p* < 0.05) with AN, AP, and selected enzyme activities (Figure S4B).

### 3.7. Stage-Dependent Changes in Carbon and Nitrogen Metabolism and Environmental Adaptation Functions of the Rhizosphere Bacterial Community

**Figure 7:**
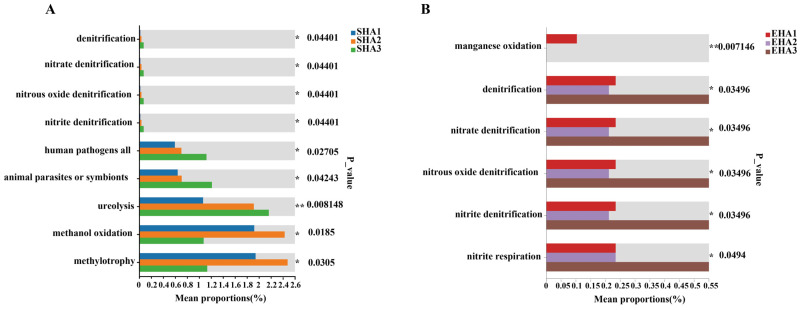
Analysis of relative abundance and statistical differences in bacterial functional pathways in the *H. annuus* rhizosphere and root endosphere based on FAPROTAX annotation. (**A**) Mean proportions (%) and corresponding *p*-values of key pathways (e.g., denitrification, human pathogens infection, animal parasitism/symbiosis) in rhizosphere samples (SHA1: seedling, SHA2: squaring, SHA3: flowering). (**B**) Mean proportions (%) and corresponding *p*-values of pathways (e.g., manganese oxidation, denitrification, nitrite respiration) in root endosphere samples (EHA1: seedling, EHA2: squaring, EHA3: flowering). Colors represent different developmental stages; *p*-values indicate differences between stages, * indicates *p* < 0.05, ** indicates *p* < 0.01.

Chemoheterotrophy and aerobic chemoheterotrophy were the most abundant predicted functional categories across all stages (Figure S5A). Several nitrogen-related functional categories, including nitrate reduction, nitrite reduction, nitrogen fixation, and denitrification, showed higher relative abundances at the flowering stage compared with earlier stages (*p* < 0.05). Among these, ureolysis- and denitrification-associated functions exhibited significant differences among developmental stages (Figure 7A).

Predicted carbon utilization functions related to methanol oxidation and methylotrophy reached their highest relative abundances at the squaring stage (SHA2)(Figure 7A, *p* < 0.05). Functions with lower overall relative abundance, including sulfur-related processes and iron respiration, also showed increased relative abundance at the flowering stage (SHA3). Functional categories annotated as potentially pathogenic or symbiotic were most abundant at the seedling stage (SHA1) and decreased at later stages.

### 3.8. Stage-Specific Variation in Carbon–Nitrogen Metabolism and Environmental Adaptation Functions of Endophytic Bacterial Communities

Chemoheterotrophy and aerobic chemoheterotrophy were the most abundant predicted functional categories across all stages. Their relative abundances were highest at the seedling stage (EHA1) and declined progressively at subsequent stages (Figure S5B). Predicted nitrogen-related functions, including nitrate reduction, nitrate respiration, and nitrogen fixation, were consistently detected at relatively high abundance throughout development, with higher relative abundance observed at the squaring (EHA2) and flowering (EHA3) stages. Predicted carbon metabolism–related functions, such as methanol oxidation and methylotrophy, were also abundant across stages but showed reduced relative abundance at the flowering stage. Kruskal–Wallis H tests identified significant differences (*p* < 0.05) among developmental stages for multiple nitrogen-related functional categories (Figure 7B), including manganese oxidation, denitrification (nitrate, nitrite, and nitrous oxide denitrification), and nitrite respiration. These functions exhibited their highest relative abundance at the flowering stage (EHA3).

## 4. Discussion

Research on crop root-associated microbiota is growing, yet the synergistic succession of bacterial communities in the rhizosphere and root endosphere across developmental stages in typical saline–alkali farmland remains poorly characterized. Existing studies often focus on single growth periods or single niches (either rhizosphere or endosphere), failing to reveal the dynamic assembly patterns of microbial communities driven by plant development, spatial niches, and saline–alkali stress combined. Particularly in agricultural regions like the Hetao Plain, characterized by irrigation-induced salinization, a critical gap exists. There is a lack of continuous, contextualized empirical data showing how the root-associated microbiome of *H. annuus*, a vital salt-tolerant cash crop, responds and structurally reorganizes across its developmental stages in this saline–alkaline environment.

This study investigated *H. annuus* growing in saline–alkali soil within the Hetao Plain. Using bacterial amplicon sequencing, we characterized compositional shifts, assembly mechanisms, and underlying functional changes in rhizosphere and root endophytic bacterial communities at seedling, squaring, and flowering stages. We provide critical evidence on the temporal (developmental stage) and spatial (rhizosphere/endosphere niche) dimensions of root microbiota succession in saline–alkaline farmland. Furthermore, we integrated microbial community changes (composition) with environmental factors, network properties, and assembly processes. This enabled us to develop an integrated ecological model depicting the dynamic reorganization of *H. annuus* root-associated microbiota under saline–alkali stress. This model offers new perspectives for understanding the stage-specific regulatory mechanisms of plant–microbe interactions.

### 4.1. Niche Differentiation of Rhizosphere and Endospheric Bacterial Communities in H. annuus Under Saline–Alkali Stress

This study systematically revealed stable and significant niche differentiation between *H. annuus* rhizosphere and root endophytic bacterial communities under real salt–alkali field conditions. Throughout the growth cycle, species richness (Chao1) and diversity (Shannon index) were consistently higher in the rhizosphere than within the roots (Figure 1A,B). This pattern is likely attributable to the rhizosphere’s role as the direct interface between soil and roots [36]. Continuous input of root exudates, characterized by highly dynamic carbon types and concentrations, provides ample energy and niche space for diverse microbial taxa [37]. In contrast, the endospheric environment forms a highly selective microhabitat. Constraints including osmotic pressure, pH, and plant immune regulation significantly limit the phylogenetic spectrum of colonizable microorganisms [12,38,39].

Saline–alkali stress likely amplifies this niche difference. High salinity and alkalinity impose stricter requirements for plant ion homeostasis and cellular integrity. This maintains strong host filtering pressure on endospheric microbiota, driving communities towards lower diversity and higher phylogenetic clustering (Figure 3) [40]. Conversely, the rhizosphere acts as a “buffer zone” between soil and roots. While affected by saline–alkali conditions, it can be locally modulated by root exudates [41,42,43], facilitating coexistence of diverse bacteria (Figure 2) [44,45]. This interplay between an “open” rhizosphere and a “constrained” endosphere establishes the foundation for divergent community succession pathways across subsequent developmental stages.

### 4.2. Stage-Specific Ecological Significance of PGPR Enrichment in the Seedling-Stage Rhizosphere

Following the confirmation of the overall differentiation pattern between rhizosphere and endosphere communities, a notable observation emerged. Bacterial taxa represented by *Pseudomonas* and *Bacillus* occupied a higher relative abundance in the *H. annuus* rhizosphere during the seedling stage (Figure 2B,D). Although this study did not directly measure their functional activities, both genera are widely reported in various crop systems as typical Plant Growth-Promoting Rhizobacteria (PGPR) [46,47]. Their stage-specific enrichment at the seedling stage possesses a strong ecological rationale.

The seedling stage is often regarded as one of the most salt–alkali-stress-sensitive periods for *H. annuus* [10,48]. At this stage, the root system is not fully developed, and the plant’s intrinsic capacity to regulate osmotic stress and ion toxicity is limited. Under these conditions, microbial taxa capable of rapid colonization and adaptation to saline–alkali environments are more likely to gain a competitive advantage in the rhizosphere [49]. Existing studies indicate that *Pseudomonas* and *Bacillus* taxa typically exhibit high environmental adaptability. They have been reported to possess diverse potential traits, including involvement in osmoregulation, extracellular polysaccharide secretion, and metabolism of phytohormones and siderophores [50,51]. Therefore, their relative enrichment in the seedling rhizosphere likely reflects an adjustment in community structure aligned with plant developmental stage. Specifically, before the plant fully establishes its own stress tolerance mechanisms, beneficial microbes may contribute to stabilizing the rhizosphere microenvironment through their stage-specific prominence [52]. This represents a shift towards potentially more beneficial community members.

It should be noted, however, that this concept of “early functional compensation” primarily constitutes an ecological inference. It is based on observed community shifts and reports from existing literature. The specific functional roles of these microbes require confirmation through subsequent isolation, cultivation, and experimental validation.

### 4.3. Developmental Stage-Driven Differentiation in Interaction Patterns and Convergence of Root Endophytic Core Microbiota

Co-occurrence network analysis revealed clear developmental stage-dependent divergence in interaction patterns between rhizosphere and root endophytic bacterial communities. Throughout *H. annuus* growth, the rhizosphere—acting as an open and continuously replenished niche—maintained relatively high network complexity, consistent with its exposure to fluctuating resource availability and ongoing microbial immigration from bulk soil [53]. Notably, a relative increase in unclassified genera was observed in the late-stage rhizosphere (Figure 2B). Rather than indicating directional host selection, this pattern likely reflects progressive diversification of available micro-niches driven by developmental shifts in root exudation and physicochemical heterogeneity under saline–alkaline stress. Such conditions may favor the persistence of taxonomically unresolved, low-abundance soil-derived taxa, which could potentially contribute to network density and interaction redundancy (Figure 4A–C, Table 1), thereby sustaining rhizosphere structural complexity despite ongoing community turnover. In contrast, the root endosphere exhibited pronounced network contraction during later developmental stages, becoming increasingly dominated by a limited set of core taxa (Figure 4D–F). This pattern reflects the strong physical and physiological constraints imposed by host tissues, which progressively channel endophytic community organization toward a centralized configuration as development proceeds [54].

Under saline–alkaline conditions, this developmental filtering was accompanied by marked convergence of the root endophytic community in terms of composition (Figure 2A and Figure 3A), interaction structure (Figure 6A,B), and predicted function (Figure 7). Notably, *Pseudomonas* showed a significant increase in relative abundance in the flowering-stage endosphere and occupied a central position in the co-occurrence network, as indicated by highly connected ASVs. At the same time, functional predictions revealed peak levels of multiple nitrogen transformation–associated pathways during this stage (Figure 7B). Together, these patterns indicate that late-stage endophytic assembly is not a random reorganization, but rather follows a structured trajectory shaped by persistent ecological filtering [55,56].

The prominence of *Pseudomonas* in both community composition and network topology suggests that its importance in the late-stage endosphere extends beyond numerical dominance. Previous studies have demonstrated that in planta inoculation with *Pseudomonas aeruginosa* PF23 and *Pseudomonas stutzeri* can alleviate salt-induced growth inhibition in *H. annuus*, underscoring the ecological relevance of *Pseudomonas* under saline–alkaline stress [57,58]. However, in the present system, the enrichment and network centrality of *Pseudomonas* are more plausibly interpreted as indicators of high adaptive fitness and structural importance within an increasingly constrained endophytic environment, rather than direct evidence of growth-promoting activity.

This interpretation is further supported by βNTI analyses (Figure S2), which indicated a shift in the root endophytic community toward drift-dominated assembly during later developmental stages. Under such assembly regimes, taxa capable of stable persistence within host-regulated internal niches are more likely to be retained and to assume central roles in microbial interaction networks. In this context, the concurrent enhancement of nitrogen-related functional potentials at the flowering stage may reflect alignment between host developmental demands—such as internal nitrogen form regulation and nutritional stability during reproductive growth—and the selective retention of ecologically compatible endophytic taxa.

Importantly, these conclusions are based on community-level associations and predictive modeling rather than direct functional measurements. While 16S rRNA gene amplicon sequencing effectively captures microbial presence, relative abundance, and inferred assembly dynamics, it cannot distinguish metabolically active microorganisms from residual DNA, nor can it establish causal functional relationships. Future studies should focus on isolating representative core taxa identified here and evaluating their stage-specific effects on *H. annuus* performance through stepwise pot and field inoculation experiments, thereby providing experimental validation for the proposed developmental stage–dependent plant–microbiome interaction model under saline–alkaline conditions.

## 5. Conclusions

This study reveals clear spatial and developmental differentiation in *H. annuus* root-associated bacterial communities under saline–alkaline field conditions. The rhizosphere maintained higher diversity and interaction complexity across growth stages, whereas the root endosphere underwent progressive convergence, driven by strong host filtering. Early stage rhizosphere enrichment of typical PGPR taxa suggests flexible microbial restructuring during stress-sensitive periods. In contrast, late-stage endophytic communities were characterized by reduced network complexity, drift-dominated assembly, and the emergence of a limited core microbiota. Notably, *Pseudomonas* became enriched and centrally positioned in endophytic networks at flowering, coinciding with increased nitrogen-related functional potential. Together, these results support a developmental stage-dependent plant–microbiome interaction model, providing a foundation for stage-targeted microbial strategies in saline–alkaline agriculture.

## Figures and Tables

**Table 1:** Network parameters of bacterial co-occurrence networks in the root endosphere and rhizosphere at different developmental stages.

Network Parameters	Endosphere			Rhizosphere		
	EHA1	EHA2	EHA3	SHA1	SHA2	SHA3
Number of nodes	102	61	66	104	123	114
Number of edges	222	51	70	229	205	297
Positive correlations	0.8693	0.8039	0.8271	0.6463	0.6	0.4882
Negative correlations	0.1307	0.1961	0.1429	0.3537	0.4	0.5118
Mean node connectivity	4.404	1.672	2.121	4.404	3.333	5.211
Modularity	0.8621	0.9438	0.9110	0.8621	0.9076	0.8639

## Data Availability

The original contributions presented in this study are included in the article/Supplementary Material. Further inquiries can be directed to the corresponding author.

## Supplementary Materials

The following supporting information can be downloaded at: https://www.mdpi.com/article/10.3390/microorganisms14020404/s1, Figure S1: Chao1 Rarefaction Curves of Sunflower Bacterial Communities Across Developmental Stages; Figure S2: Analysis of Relative Contributions of Ecological Processes in Sunflower Bacterial Communities; Figure S3: Core and Stage-Specific Bacterial Genera in Sunflower Rhizosphere and Endosphere; Figure S4: Correlations Between Dominant Genera and Soil Variables in Sunflower Rhizosphere and Endosphere; Figure S5: Predicted Functional Profiles of Sunflower Bacterial Communities. Table S1: Alpha diversity indices of bacterial communities in the rhizosphere and endosphere of sunflower across developmental stages (world file); Table S2: Statistical summary of the Redundancy Analysis (RDA) for bacterial community structure in relation to environmental factors (excel file).
