# Supplementary material for: Developmental Stage-Driven Niche Differentiation and Assembly of Rhizosphere and Endophytic Bacterial Communities in Helianthus annuus Under Saline–Alkaline Stress
Source: Microorganisms. 2026 Feb 8;14(2):404. doi: 10.3390/microorganisms14020404 (PMC12942853; doi:10.3390/microorganisms14020404)
Supplement: Supplementary file 1 [file microorganisms-14-00404-s001.zip › Supplementary data..pdf]

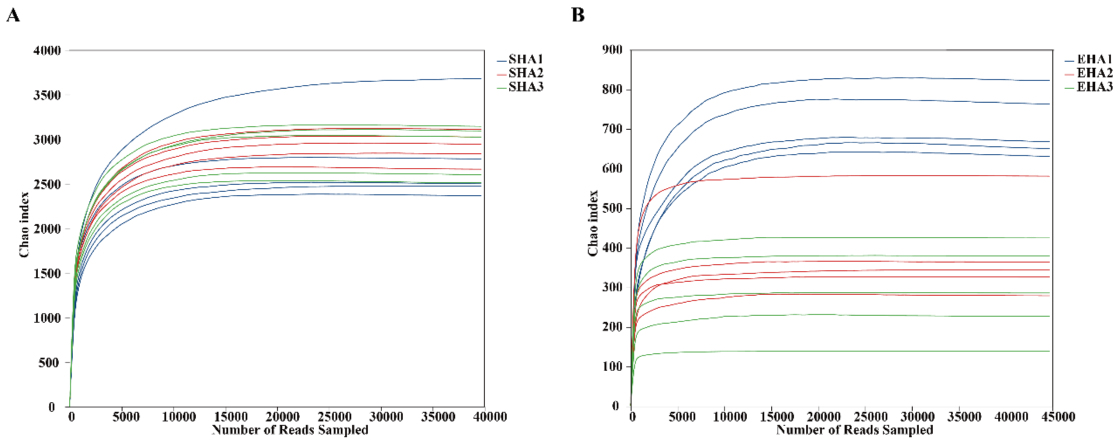

Figure S1 Chao1 rarefaction curves of sunflower bacterial communities across developmental stages. Chao1 rarefaction curves showing that sequencing depth approached saturation for both rhizosphere (A) and root endophytic (B) bacterial communities across the seedling, bud, and flowering stages of sunflower, indicating sufficient coverage to capture bacterial diversity.

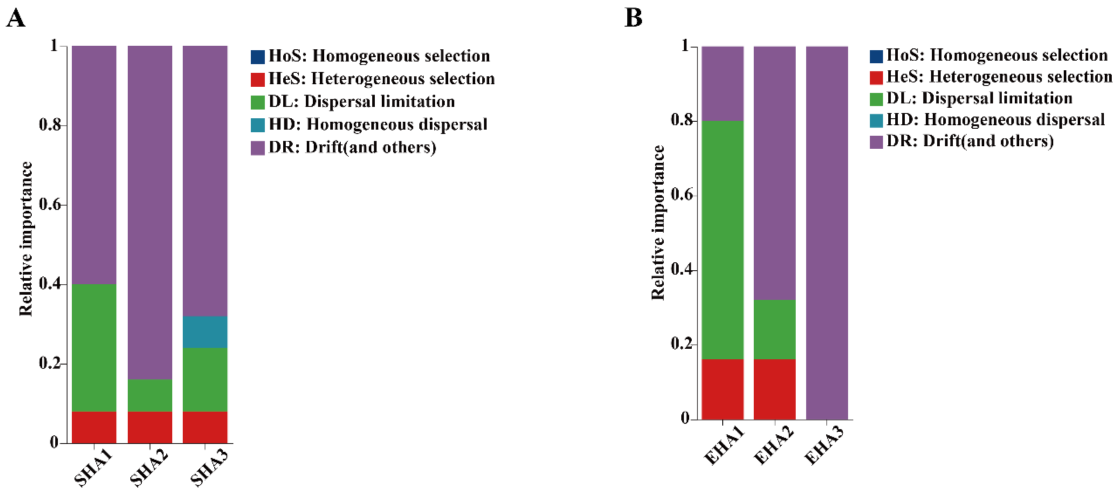

Figure S2 Analysis of relative contributions of ecological processes in sunflower bacterial communities. Relative contributions of ecological assembly processes inferred from βNTI and RC_bray analyses in rhizosphere (A) and endophytic (B) communities across developmental stages.

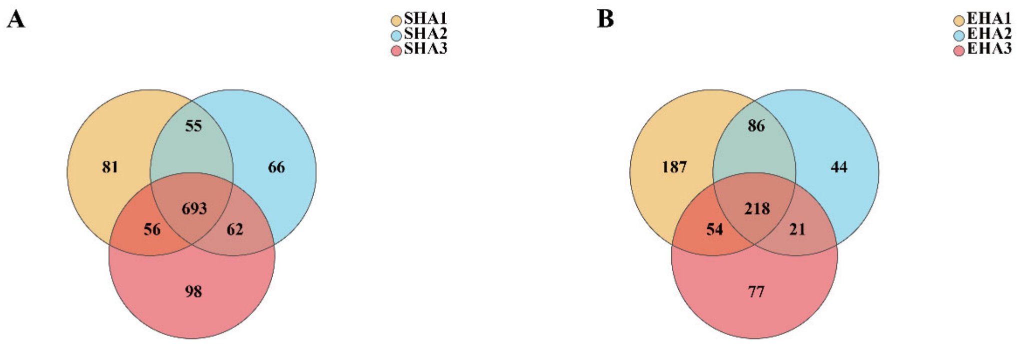

Figure S3 Core and stage-specific bacterial genera in sunflower rhizosphere and endosphere. Venn diagrams showing shared and stage-specific genera in the rhizosphere (A) and endosphere (B).

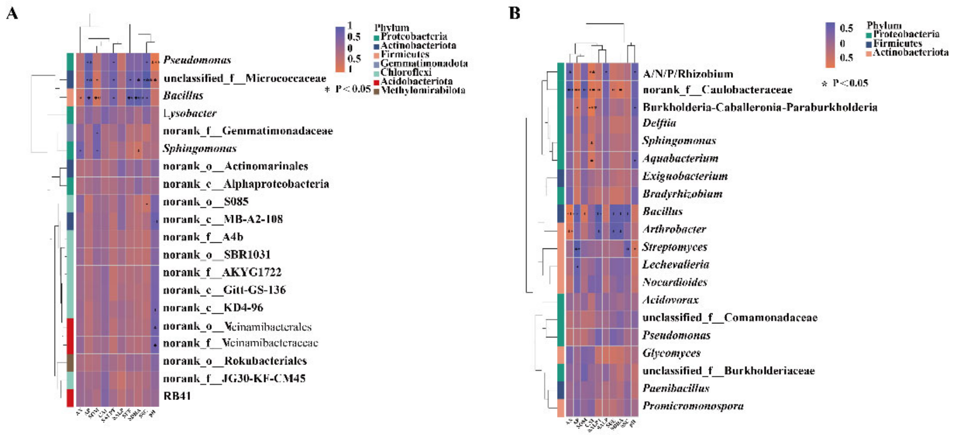

Figure S4 **Correlations between dominant genera and soil variables in sunflower rhizosphere and endosphere.** Heatmaps depict Spearman's correlation coefficients for the rhizosphere (A) and endosphere (B). Red and blue indicate positive and negative correlations, respectively; asterisks denote significance (* $p < 0.05$, ** $p < 0.01$). Phylum affiliations are shown on the side.

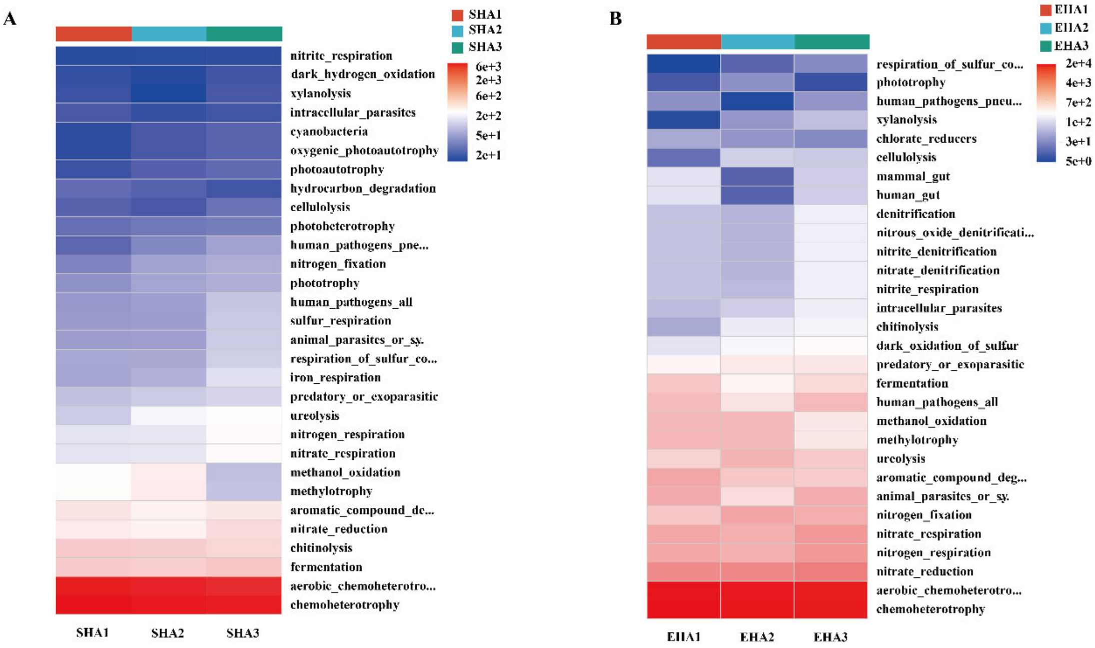

Figure S5 **Predicted functional profiles of sunflower bacterial communities.** Heatmaps show the relative abundance of predicted major functions across developmental stages in the rhizosphere (A) and endosphere (B), based on FAPROTAX annotation.

Table S1. **Alpha diversity indices of bacterial communities in the rhizosphere and endosphere of sunflowers across developmental stages.**

Alpha diversity	Rhizosphere			Endosphere		
	Sha1	Sha2	Sha3	Eha1	Eha2	Eha3
Chao1	2757.1 a	2914.1 a	2873.8 a	707.2 b	378.8 b	291.4 b
Shannon	6.8599 a	7.3272 a	7.3946 a	5.0462 b	4.5074 c	4.4359 c
